# Dementia ascertainment in India and development of nation‐specific cutoffs: A machine learning and diagnostic analysis

**DOI:** 10.1002/dad2.70049

**Published:** 2025-03-28

**Authors:** Danny Maupin, Hongxin Gao, Emma Nichols, Alden Gross, Erik Meijer, Haomiao Jin

**Affiliations:** ^1^ School of Health Sciences Faculty of Health and Medical Sciences University of Surrey, Stag Hill University Campus Guildford UK; ^2^ Center for Economic and Social Research University of Southern California VPD Los Angeles California USA; ^3^ Leonard Davis School of Gerontology Andrus Gerontology Center University of Southern California Los Angeles California USA; ^4^ Department of Epidemiology Bloomberg School of Public Health Johns Hopkins University Baltimore Maryland USA

**Keywords:** aging, assessment, cognitive decline, explainable machine learning, public health

## Abstract

**Introduction:**

Cognitive assessments are useful in ascertaining dementia but may be influenced by patient characteristics. India's distinct culture and demographics warrant investigation into population‐specific cutoffs.

**Methods:**

Data were utilized from the Longitudinal Aging Study in India‐Diagnostic Assessment of Dementia (*n* = 2528). Dementia ascertainment was conducted by an online panel. A machine learning (ML) model was trained on these classifications, with explainable artificial intelligence to assess feature importance and inform cutoffs that were assessed across demographic groups.

**Results:**

The Informant Questionnaire of Cognitive Decline in the Elderly (IQCODE) and Hindi Mini‐Mental State Examination (HMSE) were identified as the most impactful assessments with optimal cutoffs of 3.8 and 25, respectively.

**Discussion:**

An ML assessment of clinician dementia ratings identified IQCODE and HMSE to be the most impactful assessments. Optimal cutoffs of 3.8 and 25 were identified and performed excellently in the overall sample, though did decrease in specific, more difficult‐to‐diagnose subgroups.

**Highlights:**

- Pioneers use of explainable artificial intelligence in the diagnosis of dementia.
- Creates assessment cutoffs specific to the nation of India.
- Highlights differences in cutoffs across nations.

## 1 BACKGROUND

Dementia is a complex, neurodegenerative disease characterized by a combination of cognitive and functional decline. Due to increases in life expectancy, the number of people living with dementia is expected to triple by 2050, predominantly affecting middle‐ to low‐income countries.^1^ Diagnosis can be challenging, often requiring multiple medical visits across a team of health care professionals. These challenges contribute to the underdiagnosis of dementia in the community.^2^ Early diagnosis is crucial in dementia not only in terms of interventions, but also for future planning. Overcoming the difficulties of dementia diagnosis is a critical, but challenging problem, especially in middle‐ to low‐income countries with a rapidly aging population, in which individuals may have decreased ability to access medical systems.

By 2050, it is projected that nearly 20% of the total Indian population will be 60 years or older, accounting for 15.4% of all individuals over the age of 60 worldwide.^3^ The corresponding rise in dementia accompanying these changes in age structure will likely have significant ramifications on the country. Dementia has systematic adverse effects not only on individual health (e.g., disability, reduced quality of life) and family stress (e.g., significant carer burden and poor quality of life), but also on economic costs.^4^ Current research estimates the annual household cost attributable to dementia to be 571 USD, equivalent to 20% of the annual healthcare spending by the Indian government.^5^ Implementation of public health strategies, including screening, are key to not only reducing the financial costs of dementia, but also carer burden while improving health outcomes on those afflicted by the disease.^6^

A multitude of cognitive assessment tools exist and their relative utility for dementia ascertainment for older adults in India remains unclear. Two popular choices are the Informant Questionnaire of Cognitive Decline in the Elderly (IQCODE)^7^ and the Hindi‐Mental State Examination (HMSE), a modified and translated version of the Mini‐Mental State Examination.^8^ However, other potentially clinically useful tools include Instrumental Activities of Daily Living (IADL),^9^ Telephone Interview for Cognitive Status (TICS) (n.b., this does not have to be given over the phone),^10^ and the Blessed Test.^11^

The optimal cutoff of such assessments for the Indian population is under‐researched. For example, an IQCODE score of 3.9 has previously been used to classify individuals with dementia,^12^ while a score of 23 on the HMSE has been used to a similar capacity.^13^ A combined score ≥ 17 on the HMSE and < 3.35 on the IQCODE have been used to identify a sample with no literacy bias.^14^ However, the studied samples that led to these proposed cutoffs were limited in scope. The IQCODE cutoff has been previously reported as the mean score in a group of dementia patients currently utilizing dementia care services^15^ and has been used to classify individuals with a high risk of dementia.^16^ The HMSE cutoff was found in a study of 100 illiterate participants.^13^ Though appropriate when trying to remove any literacy bias, other studies have identified an ideal HMSE cutoff at 23.^17^, ^18^ This highlights the need for assessing the performance of these tools in a large, nationally representative sample that can provide stronger and more generalizable evidence on the most appropriate cutoffs for use in general samples. Additionally, studies performed in other countries have identified differing cutoffs, such as > 4.0 and < 23 or 24 on the IQCODE and MMSE, respectively, in Switzerland,^19^ or IQCODE cutoffs of 3.38 previously identified in England.^20^

The use of multiple assessments for dementia screening may be appropriate given its hallmark declines in both cognitive and functional capabilities. The combination of tools that assess both characteristics will likely lead to an improved and more consistent ascertainment tool. Though the combination of one or more tools together may result in greater accuracy (i.e., closer to correct case‐ness) than one tool alone,^19^ it is unclear how optimal cutoffs may be impacted when assessments are used together.

To address these gaps, our current research has multiple goals. First, we compare the importance of a variety of cognitive and functional assessments to dementia ascertainment and identify the strongest contributors to machine learning (ML) outcomes trained on a nationally representative sample from India. Second, we will examine the optimal cutoffs of identified assessments for the Indian population. Last, performance of the optimal cutoffs will be assessed across a range of differing demographic groups to ensure consistent performance and minimal bias.

**RESEARCH IN CONTEXT**

1. **Systematic review**: The authors reviewed literature using traditional sources (e.g., PubMed). The previous results using these cutoffs in Indian populations are consistent with this paper's results, while variation exists within other nations.
2. **Interpretation**: Our findings indicate that cutoffs may vary across different nations, necessitating research not only into optimal cutoffs for dementia identification but also consideration of the social and demographic factors that can affect assessment results
3. **Future directions**: This article provides a path for future research into nation‐specific cutoffs, while accounting for social and demographic factors. Further, it is pioneering the use of explainable artificial intelligence methodologies to provide further clarity and openness into machine learning models, options that can continue to be explored in this field.

## 2 METHODS

### 2.1 Participants

The Longitudinal Aging Study in India—Diagnostic Assessment of Dementia (LASI‐DAD) is a sub‐study of the Longitudinal Aging Study in India (LASI).^16^ Between 2018 and 2020, a subsample of age eligible (60+) LASI respondents were recruited for an in‐depth dementia assessment and clinical ascertainment (*N* = 4096). A two‐stage stratified random sampling approach that oversampled respondents at a higher risk of cognitive impairment was used to maintain national representation and ensure a sufficient number of respondents with cognitive impairment. Risk of cognitive impairment was based on the cognitive tests in the main LASI study and proxy reports for those who could not complete the cognitive tests during the LASI study. Clinical ascertainment of dementia was obtained through an online consensus process for 2528 (61.7%) LASI‐DAD participants.^21^ We used data from all participants who underwent clinical dementia ascertainment. LASI‐DAD data were collected in three phases. The participants in phases 2 and 3 were clinically assessed. Which phase an individual was interviewed in depended on fieldwork logistical considerations, primarily the state of residence, and not on any individual characteristics such as cognitive status. Therefore, clinical assessment is likely missing at random, and we would not expect selection bias because of this. Data were accessed from the Gateway to Global Aging Data website.^22^

### 2.2 Factors used in dementia assessments

The variables included in the Clinical Dementia Rating (CDR) assessment have previously been described,^21^ and the full list is provided in File S1. Six categories were provided to clinicians, including cognitive assessment, self‐reported functional difficulties, depression and anxiety, informant interview, sociodemographic variables, and health history. The cognitive assessment included HMSE,^8^ TICS,^10^ Community Screening Instrument for Dementia,^23^ numeracy problems,^24^ and judgment and problem‐solving questions.^25^ Self‐reported functional difficulties consisted of Activities of Daily Living (ADL)^26^ (e.g., dressing, walking) and IADLs^9^ (e.g., preparing a meal, shopping for groceries). Depression and anxiety consisted of the Beck Anxiety Inventory^27^ and 10‐item Center for Epidemiological Studies Depression Scale.^28^ Assessments provided by the informant interview included IQCODE,^7^ and the Blessed Test.^11^ Sociodemographic variables (e.g., age, sex) and health history (e.g., previous history of dementia, stroke, diabetes, and other health conditions) were provided. Missing data on cognitive tests and informant reports were imputed following the procedure outlined by the LASI‐DAD codebook Version A.3, 2022. Briefly, a regression model was used to specify a conditional distribution for each cognition variable with imputations calculated from pseudorandom draws from this conditional distribution. Previous research in LASI‐DAD focused on the IQCODE showed that use of imputations did not substantively impact study conclusions.^29^

### 2.3 Dementia ascertainment

Dementia status was determined via a consensus Web‐based panel using the CDR.^25^ The methodology of this process has previously been described in depth^30^ and is briefly introduced here. Clinical researchers train non‐clinician research interviewers to obtain key information through a battery of cognitive assessments for respondents and informants in a manner that addresses the key issues in the CDR. Data from each respondent are reviewed by three clinicians, who individually gave a CDR rating based on the standardized data. When there are disagreements in individual ratings, cases are reviewed by a panel of clinicians. If full consensus was not reached, a majority rating was recorded, with a flag indicating the persistent differences in CDR. This process has previously been shown to demonstrate a high consistency rate of 90.8% with an in‐person clinical assessment, and excellent inter‐rater agreement between online consensus and in‐person clinical diagnosis (kappa = 0.75).^31^ Participants with a consensus CDR score of 1 and above were classified as having dementia.

### 2.4 ML and statistical analysis

As described above, dementia was ascertained based on a battery of various cognitive assessments through a clinical consensus process. A ML model was trained to predict dementia status based on the same variables presented to the clinical researchers when conducting the clinical consensus process. Factor importance was assessed using an explainable artificial intelligence tool known as SHapley Additive exPlanations (SHAP). This process provides an overview of each features’ mean contribution to the ML model's outcomes. This process is detailed in full below. Additionally, to provide further context, independent *t*‐tests were performed between those that were assessed with dementia and those that were assessed as cognitively normal. Variables of interest included demographic information, such as age and gender, cognitive tests such as the HMSE, and functional tests, such as ADLs.

#### 2.4.1 ML Model

We utilized an eXtreme Gradient Boosting (XGBoost) ML algorithm to predict dementia ascertainment based on the variables available to clinicians at time of rating, constructed using the XGBoost package^32^ from Python. XGBoost is an optimization model consisting of both a linear and a boosting tree model resulting in improved efficiency. Additionally, in comparison to other ML algorithms, previous research has suggested that estimating SHAP values with XGBoost and similar tree‐based models is more accurate than other ML algorithms including logistic regression and support vector machines.^33^ The outcome of this model was the overall CDR rating converted to a binary outcome where 0 indicates no dementia (CDR scores of 0 and 0.5) and 1 indicates dementia (CDR scores of 1, 2, and 3).

Data were split into test and training samples using a 30/70 split and stratified on dementia status. To improve generalizability of the model and decrease the risk of overfitting, a 10‐fold cross‐validation methodology was implemented whereby the training data are split into 10 equal groups, with 1 group held out to validate the model during the training process. Hyperparameter optimization was completed using a Bayesian optimization process which involves setting a prior for the optimization function and iteratively updating this over samples.^34^ A utility function is then implemented to identify the next sample for further evaluation and updating of the optimization. The features of this model were limited in scope to the information made available to the clinicians during the dementia ascertainment process. A model was constructed for all clinicians, as well as individual clinicians who provided ratings for > 300 respondents to contrast models trained on individual datasets compared to the collective model. Nine out of 13 clinicians met this cutoff. Given the class imbalance between dementia and non‐dementia cases, a matched dataset was constructed using the MatchIt package in R.^35^ This process matched participants on the basis of age, education, marriage status, literacy, and rural living, resulting in a sample of 384 matched participants.

#### 2.4.2 Factor importance

The variables chosen for the screening tool were based off a feature selection method known as SHAP. SHAP values assess the marginal contribution value of each feature to the outcome, thereby providing a measure of feature importance.^36^ This is achieved by attributing to each feature the change in the expected model prediction when conditioning on the feature.^36^ In this paper, the calculation of SHAP values was used to identify the variables that had the largest impact, or marginal contribution, to our outcome (i.e., dementia ascertainment). SHAP values were calculated using the TreeExplainer function from the SHAP package^36^ available in Python. Calculations occurred during the cross‐validation phase of the ML model to increase sample size and present a consistent overview of variable importance. The resulting code for both the ML model and calculation of SHAP values has been uploaded online to facilitate reproducibility.^37^

#### 2.4.3 Cutoff evaluation

An iterative approach was taken to calculate the optimal cutoffs for the identified important factors. This analysis aims to produce national‐specific cutoffs of the cognitive assessments deemed important in the prior ML analysis. When multiple important factors were identified, cutoffs were used in conjunction, with participants meeting all cutoffs being classified as having dementia and those who did not meet one or more cutoffs being classified as non‐dementia. The following statistics were calculated by comparing the cutoffs against the dementia ascertainment in LASI‐DAD: accuracy (number of correct predictions divided by number of all predictions), sensitivity (number of true positives divided by the sum of true positives and false negatives), specificity (number of true negatives divided by the sum true negatives and false positives), and area under the curve (calculated area under the curve created by plotting the true positive and false positive rates across different thresholds) (AUC). Youden's Index was used as the final comparator. Youden's Index is calculated as:

$${\mathrm{Youden}}^{\prime }s\mathrm{Index}=\left(\mathrm{Sensitivity}+\mathrm{Specificity}\right)-1$$

Youden's Index was chosen to be the final comparison metric because it provides equal weights to sensitivity and specificity.^38^, ^39^ A 95% confidence interval (CI) was calculated for the AUC, sensitivity, and specificity using the pROC package in R. The 95% CI for Youden's Index were calculated using the equation defined by Chen et al.^40^ which accounts for the contingency correlation between sensitivity and specificity. This process was repeated across different demographic sub‐groups, including such as rural/urban living, low education (defined as less than 6 years of education), and illiterate respondents.

## 3 RESULTS

### 3.1 Participants

Demographic and cognitive data for the participants with dementia ascertainment in the LASI‐DAD (*n* = 2528) can be found in Table 1.

**TABLE 1:** Demographic characteristics and cognitive performance for included participants.

Demographic characteristics	Mean (SD) or N (%)
Age (years), mean (SD)	69.6 (7.5)
Female, n (%)	1324 (52%)
Years of education, mean (SD)	3.6 (4.6)
Literate, n (%)	1030 (41%)
Married, n (%)	1672 (66%)
Living in rural environment, n (%)	1655 (65%)
Cognitive assessments	Mean (SD) or N (%)
HMSE overall score, mean (SD)	22.4 (5.6)
Blessed test part 1 overall score, mean (SD)	1.3 (1.7)
Blessed test part 2 overall score, mean (SD)	1.1 (0.3)
IQCODE overall score, mean (SD)	3.5 (0.6)
TICS overall score, mean (SD)	2.0 (0.9)
Some difficulty with ADLs overall score, mean (SD)	1.3 (1.8)
Some difficulty with IADLs overall score, mean (SD)	2.3 (2.2)
***CDR final classification, n (%)***	
0	768 (30%)
0.5	1568 (62%)
1	162 (6%)
2	25 (1%)
3	5 (0.2%)

Abbreviations: ADLS, Activities of Daily Living; CDR, Clinical Dementia Rating; HMSE, Hindi Mental State Examination; IADLs, Instrumental Activities of Daily Living; IQCODE, Informant Questionnaire on Cognitive Decline in the Elderly; SD, standard deviation; TICS, Telephone Interview for Cognitive Status.

**FIGURE 1:**
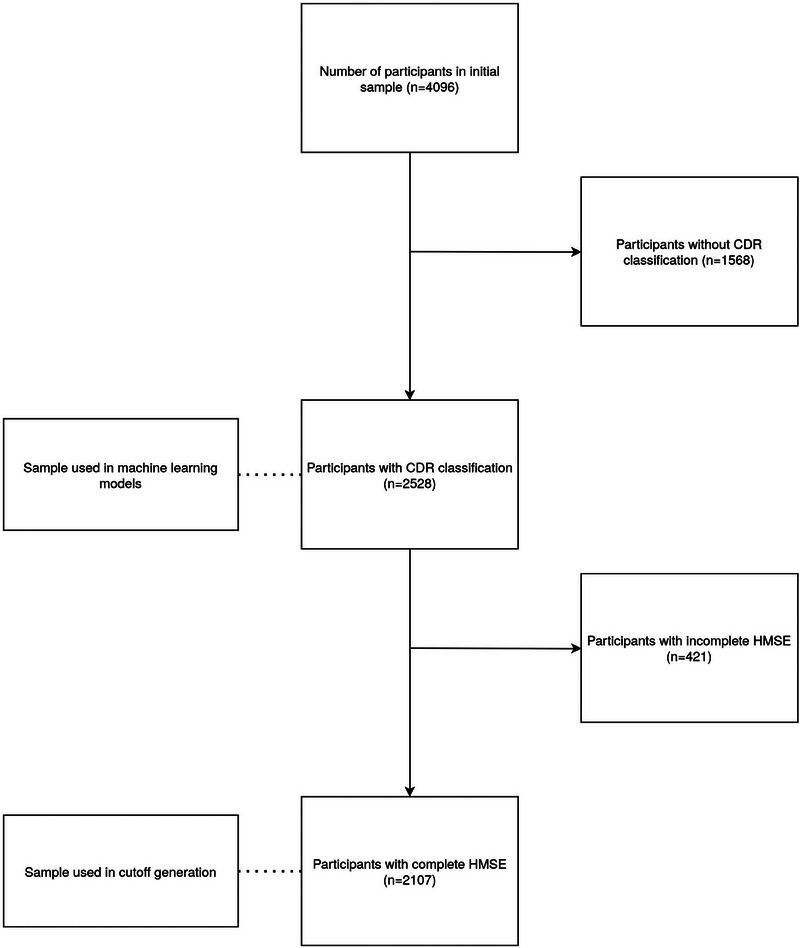
Flow chart depicting data cleaning process of included participants.

A participant flow diagram that outlines the data cleaning process can be seen in Figure 1, highlighting the number of participants used in the ML model and subsequent cutoff generation.

Results of the *t*‐tests comparing those assessed with dementia and those assessed as cognitively normal can be found in File S2.

### 3.2 ML model

The ML model, when performed on the overall dataset, demonstrated excellent performance with an AUC of 0.92 (Table 2) on a test dataset. Performance scores were found to be at least adequate, if not excellent, when the model training, validation, and testing was performed across individual clinicians (AUC ranging from 0.75 to 0.96). These demonstrate the excellent ability of the ML model to accurately predict dementia status.

**TABLE 2:** Performance metrics from the machine learning model.

Metric	Result
Accuracy	0.92
Sensitivity	0.88
Specificity	0.89
Area under the curve	0.92
Youden's index^a^	0.77

^a^ Defined as (Sensitivity + Specificity) ‐ 1. Chosen Probability Threshold: 0.11.

### 3.3 Factor importance

**FIGURE 2:**
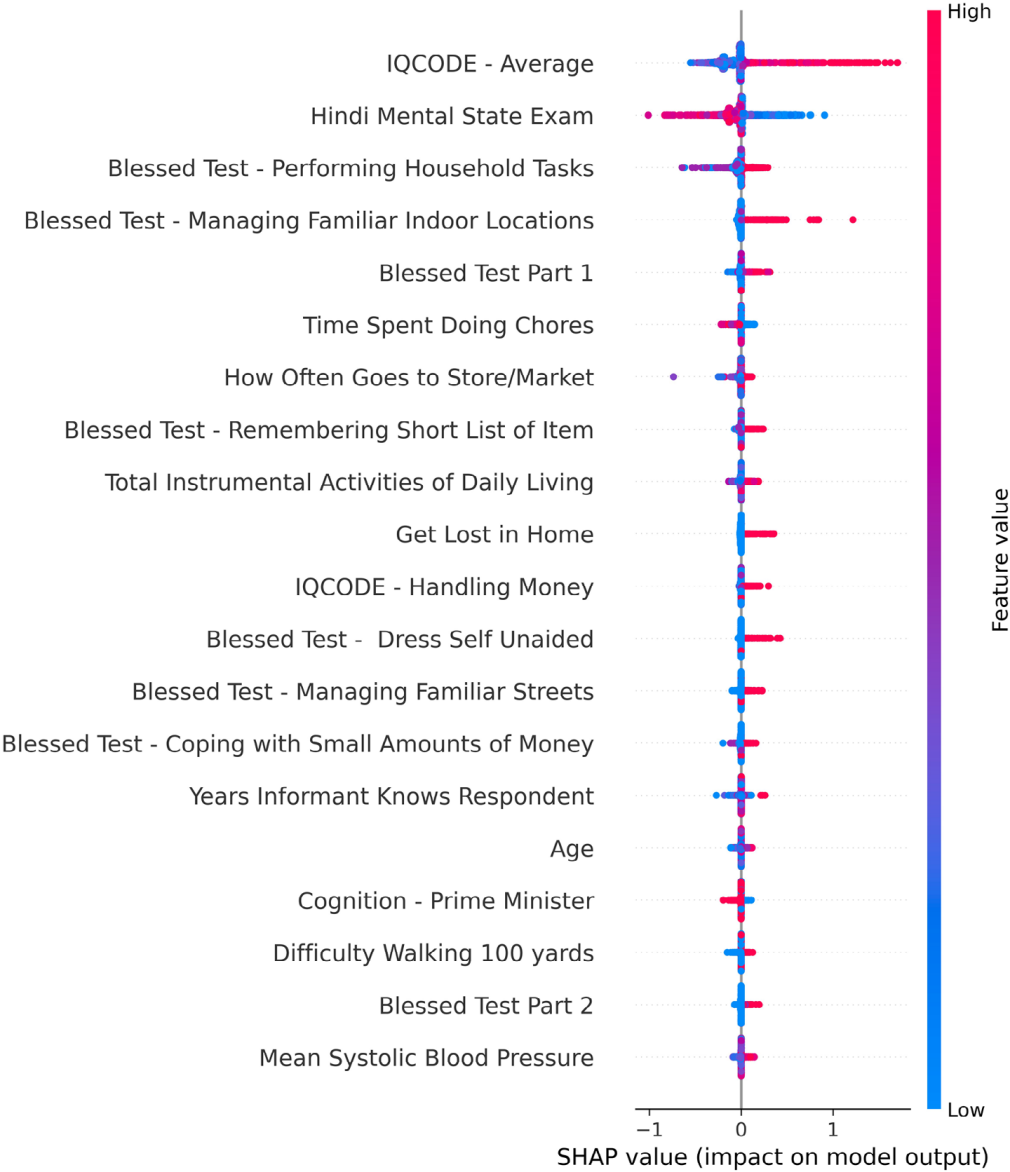
Beeswarm plot depicting feature importance as measured by Shapley Additive exPlanation values (SHAP). IQCODE, Informant Questionnaire of Cognitive Decline in the Elderly.

The two most impactful variables as measured by mean SHAP value, or average impact on model output magnitude, were the IQCODE and HMSE (Figure 2). Across the ML models trained on individual clinician datasets, variable importance varied; however, IQCODE and HMSE consistently remained important predictors (File S3). Two out of nine ML models had a combination of IQCODE and HMSE as the top two variables. Five models had IQCODE as the top variable, two had HMSE or Blessed Test Part 1 each as the top contributing variable, though the Blessed Test Part 1 did not appear in the top 20 for three out of the nine datasets. Conversely, IQCODE and HMSE were present in all nine. The IQCODE had an average rank of 1.8, while the HMSE had an average rank of 4.2. Results of the ML model on the matched sample were similar with an AUC of 0.93. The average IQCODE score remained the most impactful contributor on the basis of SHAP values while HMSE dropped to fifth most impactful. Considering the overall dataset reflects the real‐world imbalance on dementia, we proceeded with average IQCODE and HMSE as the most impactful variables.

### 3.4 Optimal cutoffs

Due to their consistent importance, IQCODE and HMSE were chosen in the calculation of optimal cutoffs. Although imputed HMSE scores were used in the prior ML analysis above, only participants with fully completed HMSE (*n* = 2107) were included in this assessment of optimal cutoffs to minimize the potential bias due to imputation. Analysis on the imputed dataset is shown in File S4. The derived cutoffs demonstrated excellent performance. The cutoffs of > 3.8 on the IQCODE and < 25 on the HMSE resulted in optimal performance on the Youden Index. These cutoff points resulted in an overall accuracy of 0.85, sensitivity of 0.90 (95% CI 0.85–0.95), specificity of 0.85 (95% CI 0.83–0.86), and Youden's Index of 0.75 (95% CI 0.71–0.78). Results remained similar when accounting for participant survey weights with an overall accuracy of 0.86, sensitivity of 0.89 (95%CI 0.83–0.94), specificity of 0.86 (95% CI 0.84–0.87), and Youden's Index of 0.74 (95% CI 0.70–0.78). The results of these thresholds on the imputed dataset show a Youden's Index of 0.67 (95% CI 0.62–0.72). Differences are present between the point estimates of the overall and imputed datasets though with overlap in the 95% CIs. The difference in point estimates may be due to sample selection with the non‐imputed data having fewer relative dementia cases compared to imputed data. Table 3 shows the confusion matrix comparing the results of these cutoffs and the original clinical dementia ascertainment in LASI‐DAD.

**TABLE 3:** 2×2 table of the optimal cutoffs for identified important cognitive assessments.

Clinical Dementia Ascertainment in LASI‐DAD		
	**Non‐dementia**	**Dementia**
**Non‐dementia**	1683	12
**Dementia**	301	111

*Note*: Optimal cutoffs: > 3.8 Informant Questionnaire on Cognitive Decline in the Elderly (IQCODE) and < 25 Hindi Mini‐Mental State Examination (HMSE).

Abbreviation: LASI‐DAD, Longitudinal Aging Study in India—Diagnostic Assessment of Dementia.

There was a subtle difference when considering the cutoff for HMSE and IQCODE individually, compared to their cutoff values when the two assessment tools are used together (as evaluated above). The cutoff for HMSE, when used individually, was 20 with an overall accuracy of 0.78, sensitivity of 0.81 (95% CI 0.74–0.88), specificity of 0.77 (95% CI 0.76–0.79), and Youden's Index of 0.59 (95% CI 0.55–0.62). The optimal IQCODE cutoff when used individually remained at 3.8 with an identified accuracy of 0.79, sensitivity of 0.93 (95% CI 0.88–0.97), specificity of 0.78 (95% CI 0.76–0.80), and Youden's Index of 0.70 (95% CI 0.68–0.73).

As shown in Table 3, the optimal cutoffs led to 12 false negatives but 301 false positives. Of these 301 individuals, 299 had a CDR rating of 0.5, suggesting that these participants potentially had signs of mild cognitive impairment that could develop into dementia. Positive results on this screening are likely to infer at least mild cognitive decline warranting further evaluation and monitoring by a specialist.

To account for the diversity of the Indian population, with heterogeneity in education, literacy, and urban/rural residence, the cutoffs’ performance was further evaluated in these subgroups (Table 4). Though the best‐performing cutoffs differed from the overall cutoffs of > 3.8 on the IQCODE and < 25 on the HMSE, these values still demonstrated similar performance, suggesting adequate generalization. The associated SHAP value plots and total metrics can be found in File S5.

**TABLE 4:** Performance of subgroup specific cutoffs and overall optimal cutoffs on different subgroups.

Subgroup	Subgroup specific cutoffs	Youden's index (subgroup specific cutoffs)	Youden's index (overall optimal cutoffs)
Female	IQCODE > 3.8, HMSE < 25^*^	0.73	0.73
Male	IQCODE > 3.8, HMSE < 26	0.77	0.77
High education (≥ 6 years)	IQCODE > 3.7, HMSE < 24	0.87	0.87
Low education (< 6 years)	IQCODE > 3.8, HMSE < 23	0.70	0.68
Illiterate	IQCODE > 3.8, HMSE < 23	0.68	0.67
Literate	IQCODE > 3.8, HMSE < 25^*^	0.89	0.89
Rural	IQCODE > 3.8, HMSE < 23	0.73	0.70
Urban	IQCODE > 3.8, HMSE < 25^*^	0.85	0.85

*Note*: Overall optimal cutoff = IQCODE > 3.8, HMSE < 25.

Abbreviations: HMSE, Hindi Mini‐Mental State Examination; IQCODE, Informant Questionnaire of Dementia in Elderly.

* Sub‐demographic cutoff is the same as overall optimal cutoff.

## 4 DISCUSSION

The goals of this article were to identify cognitive assessments that are important for making clinical ascertainment of dementia in India, as well as defining their optimal cutoffs, based on a nationally representative study sample from the LASI‐DAD study. Two cognitive assessments, IQCODE and HMSE, were identified as the most strongly impactful in a ML model's interpretation of clinician ratings, with IQCODE having a greater impact due to its higher average rank. These assessments have previously been utilized as tools for dementia ascertainment and screening throughout India.^12^, ^13^, ^15^, ^16^ Optimal cutoffs were also identified in this paper, namely > 3.8 on the IQCODE and < 25 on the HMSE.

The optimal cutoffs identified in this analysis are similar to previous research conducted in India with only slight differences. For example, a cutoff value of 3.9 has been used for the IQCODE previously in the literature.^12^, ^16^ Further, the HMSE has been used with a cutoff of 23, identified from a small sample of elderly illiterate participants.^13^ Our analysis also showed that the optimal cutoffs are similar when IQCODE and HMSE are used individually versus together. The proposed cutoffs identified in this paper are from a national, representative database that are likely more generalizable for the Indian population.

Evidence is sparse for comparison of the cutoffs for IQCODE and MMSE used together in other nations. A study of participants based in Switzerland found the best performing combined cutoff for the IQCODE and MMSE to be > 4.0 and < 23 or 24, respectively,^19^ supporting the idea that cutoffs may not be generalizable across countries, cultures, and languages. When comparing the IQCODE cutoff (3.8) on its own, the differences become even more pronounced. Previous research has used various IQCODE cutoffs to signify dementia, such as 3.38 in the English Longitudinal Study of Ageing,^20^ or 3.4 in the Mexican Health and Aging Study.^41^ The HMSE cutoff score of 25 was more in line with previously used cutoffs of 23.^17^, ^18^ Both 23^42^, ^43^ and 24^42^ are commonly used as cutoffs for the MMSE in other countries.

Screening for dementia is more challenging in particular demographic subgroups, such as those from rural, low‐education, and/or illiterate backgrounds.^44^, ^45^, ^46^, ^47^ Identified optimal cutoffs had varied performance across subgroups. While the overall performance of the identified cutoffs remained adequate, there was a meaningful depreciation in performance despite IQCODE and HMSE remaining clinically important assessment tools, as identified by SHAP values, in these subgroups (File S4). Given the depreciation, these subgroups may still remain challenging to screen. However, given the overall consistency of the scores (Youden's Index ≥ 0.73 across all groups), this cutoff is likely to still perform reasonably well across all populations.

Overall, this article has identified optimal cutoffs for the IQCODE and HMSE using a nationwide, representative dataset. These cutoffs can be implemented in a clinical or nonclinical setting, such as primary care and community‐based initiative, to assess or screen individuals for dementia in India. Further, the performance of a wider range of cutoffs is available in the supplementary files (File S6) and may be used to assess sensitivity and specificity for different cutoff values. As our approach used an outcome that balances sensitivity and specificity, a cutoff that implements a greater specificity, therefore reducing false negatives, may be more desirable in certain contexts. Additionally, researchers can use these cutoffs to classify their participants with dementia in India if they do not have access to clinicians to provide a dementia ascertainment or a larger battery or neurocognitive assessments, either of which can be costly in terms of both time and financial requirements.

One limitation in this article is possible bias in the results due to the design of the LASI‐DAD clinical consensus system potentially placing more emphasis on the IQCODE and HMSE assessments. Specifically, these two assessments, and their individual items, constitute a significant portion of the cognitive assessment section. The perceived importance of these assessments may be due to the large number of items rather than a physiological explanation. Clinicians’ viewpoints may also be contributing to the importance of these two items (i.e., IQCODE and HMSE) as they are often implemented in clinical settings. Further, though it is not surprising that IQCODE and HMSE, given their clinical utility and the LASI‐DAD design, were impactful variables in the ML model, these results should not be used to explain the decision‐making process of the clinicians involved in the LASI‐DAD project. These variables were simply the most impactful variables to the outcomes of a ML model trained on the data presented to the clinicians.

Though this article has identified that use of the IQCODE (> 3.8) and HMSE (< 25) together provides excellent ascertainment of dementia, this is not without drawbacks. The use of these two tools in combination may require a significant time investment on the part of clinicians. Further, it will require the participation of both the individual being assessed (HMSE) as well as an informant (IQCODE), necessitating further time and resource costs. Lastly, not all individuals, regardless of dementia status, are able to complete the HMSE due to a variety of reasons including literacy or education. In these cases, additional assessment tools may be required (such as the Blessed Test which is informant‐based in LASI‐DAD). The code used to generate cutoff values has been published to allow interested parties to compare assessments and their combinations. This will allow individuals to gauge assessments that may have more utility for their specific context. Future research is required to assess strong ascertainment tools without the use of HMSE, as well as potential informant or respondent only ascertainment tools to decrease the associated time and resource costs. It should be noted our analysis shows that using the IQCODE alone did result in adequate performance and the addition of other assessments (e.g., Blessed Test Part 1, IADLs) resulted in no improvement when compared to IQCODE on its own. Additionally, though the ML algorithm identified the IQCODE and HMSE as two of the more clinically important factors in this population, it is unclear if this will be true in other populations or countries. Future research will be needed to see if this is reproducible across other countries and populations.

In conclusion, dementia is a growing public health concern, particularly in low‐ to middle‐income countries such as India. Diagnosing and screening for dementia remains a complex endeavor due to the nature of the disease, and a variety of assessment tools available. Based on an explainable ML algorithm of CDR scores, two variables were found to be significantly important in determining outcomes, IQCODE and HMSE. Further evaluation based on a nationally representative dataset of India identified optimal cutoffs for these assessments when used together. These cutoff values could be used as a research or clinical tool to improve dementia screening.

## CONFLICT OF INTEREST STATEMENT

The authors declare no conflicts of interest. Author disclosures are available in the supporting information.

## CONSENT STATEMENT

Consent was not necessary for the undertaking of this research. All participants previously provided consent as part of the Longitudinal Ageing Study of India and its sub‐study Longitudinal Ageing Study of India—Diagnostic Assessment of Dementia.

## Supporting information



Supporting Information

Supporting Information

Supporting Information

Supporting Information

Supporting Information

Supporting Information

Supporting Information
