# Supplementary material for: Dementia ascertainment in India and development of nation‐specific cutoffs: A machine learning and diagnostic analysis
Source: Alzheimers Dement (Amst). 2025 Mar 28;17(1):e70049. doi: 10.1002/dad2.70049 (PMC11952995; doi:10.1002/dad2.70049)
Supplement: Supplementary file 3 — Supporting Information [file DAD2-17-e70049-s002.docx]

Supplementary Table 2: Results of t-tests comparing those classified as dementia and non-dementia

| Variable | Mean Group 1^1^ | Mean Group 2^2^ | Mean-Difference | *p*-value |
| --- | --- | --- | --- | --- |
| Age | 69.1 | 75.2 | -6.1 | < 0.001 |
| Years of Education | 3.8 | 1.3 | 2.5 | < 0.001 |
| Gender ^3^ | 1.5 | 1.6 | -0.1 | < 0.001 |
| Literacy ^4^ | 0.5 | 0.8 | -0.3 | < 0.001 |
| Rural Living ^5^ | 0.6 | 0.7 | -0.1 | 0.006 |
| HMSE | 23.0 | 14.3 | 8.7 | < 0.001 |
| Blessed Test Part 1 | 1.1 | 4.0 | -2.9 | < 0.001 |
| Blessed Test Part 2 | 1.1 | 1.5 | -0.4 | < 0.001 |
| IQCODE | 3.4 | 4.5 | -1.0 | < 0.001 |
| TICS | 2.1 | 1.1 | 1.0 | < 0.001 |
| ADLs | 1.2 | 2.9 | -1.7 | < 0.001 |
| IADLs | 2.1 | 5.0 | -3.0 | < 0.001 |

Key: ^1^Group 1: Classified as non-dementia; ^2^Group 2: Classified as dementia; ^3^Gender: 1 indicates males, 2 indicates female; ^4^Literacy: 0 indicates literate, 1 indicates illiterate; ^5^Rural Living: 0 indicates urban, 1 indicates rural; HMSE: Hindi-Mental State Examination; IQCODE: Informant Questionnaire on Cognitive Decline in the Elderly; TICS: Telephone Interview for Cognitive Status; ADL: Activities of Daily Living; IADLS: Instrumental Activities of Daily Living

Supplementary File Legends

Supplemental File 1 -- List of machine learning model variables.

Supplemental File 2 -- T-tests results across participants.

Supplemental File 3 -- SHAP values across clinicians.

Supplemental File 4 -- Cutoff performance across subgroups.

Supplemental File 5 -- SHAP values across subgroups.

Supplemental File 6 -- Table of all cutoff values.
