# Supplementary material for: Dementia ascertainment in India and development of nation‐specific cutoffs: A machine learning and diagnostic analysis
Source: Alzheimers Dement (Amst). 2025 Mar 28;17(1):e70049. doi: 10.1002/dad2.70049 (PMC11952995; doi:10.1002/dad2.70049)
Supplement: Supplementary file 4 — Supporting Information [file DAD2-17-e70049-s001.docx]

Supplemental File 3: Figures show beeswarm plots depicting variable importance as measured by SHAP (Shapley Additive Explanations) values for participants subset by different raters. For example, Rater 1 shows the variable importance for all participants assessed by Rater 1.

Rater 1:


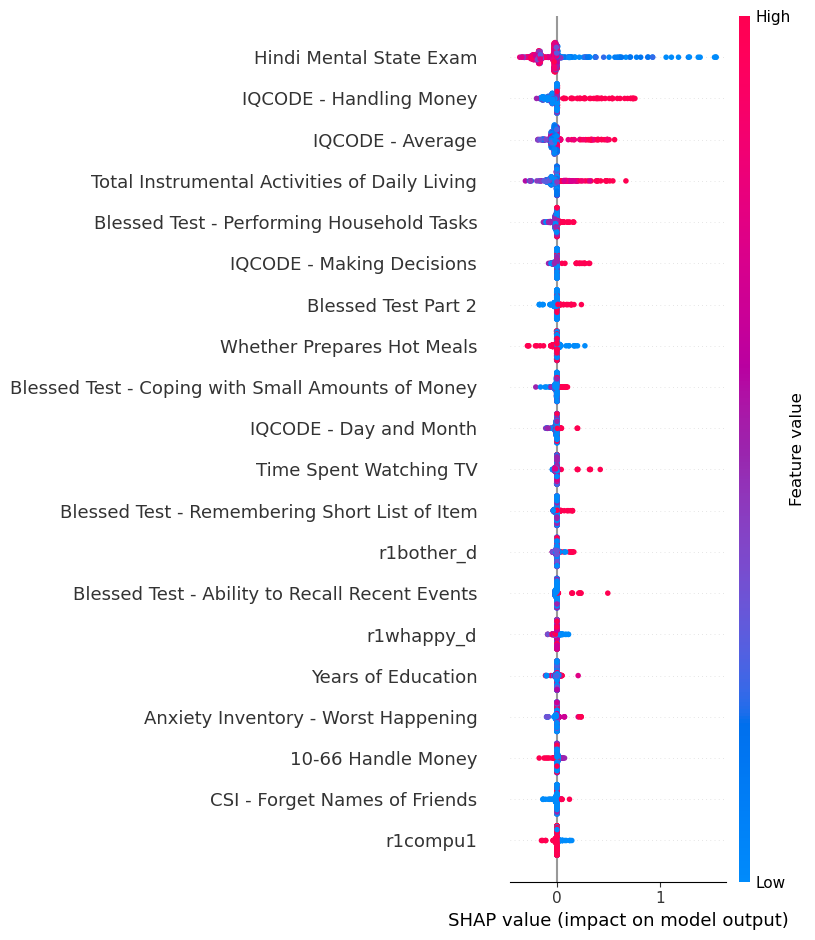


Threshold to Maximise Youden’s Index = 0.11

| Metric | Result |
| --- | --- |
| Accuracy | 0.93 |
| Sensitivity | 0.88 |
| Specificity | 0.94 |
| Area Under the Curve | 0.96 |
| Youden’s Index* | 0.82 |

* Defined as (Sensitivity + Specificity) - 1

Rater 2


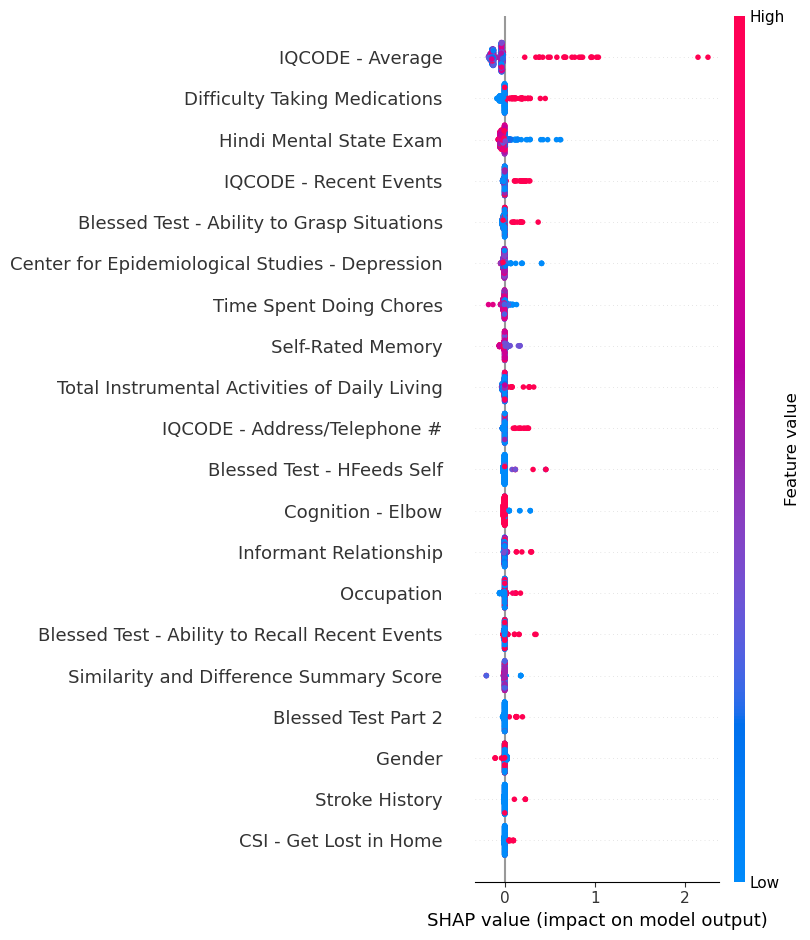


Threshold to Maximise Youden’s Index = 0.08

| Metric | Result |
| --- | --- |
| Accuracy | 0.88 |
| Sensitivity | 0.75 |
| Specificity | 0.89 |
| Area Under the Curve | 0.84 |
| Youden’s Index* | 0.64 |

* Defined as (Sensitivity + Specificity) - 1

Rater 3


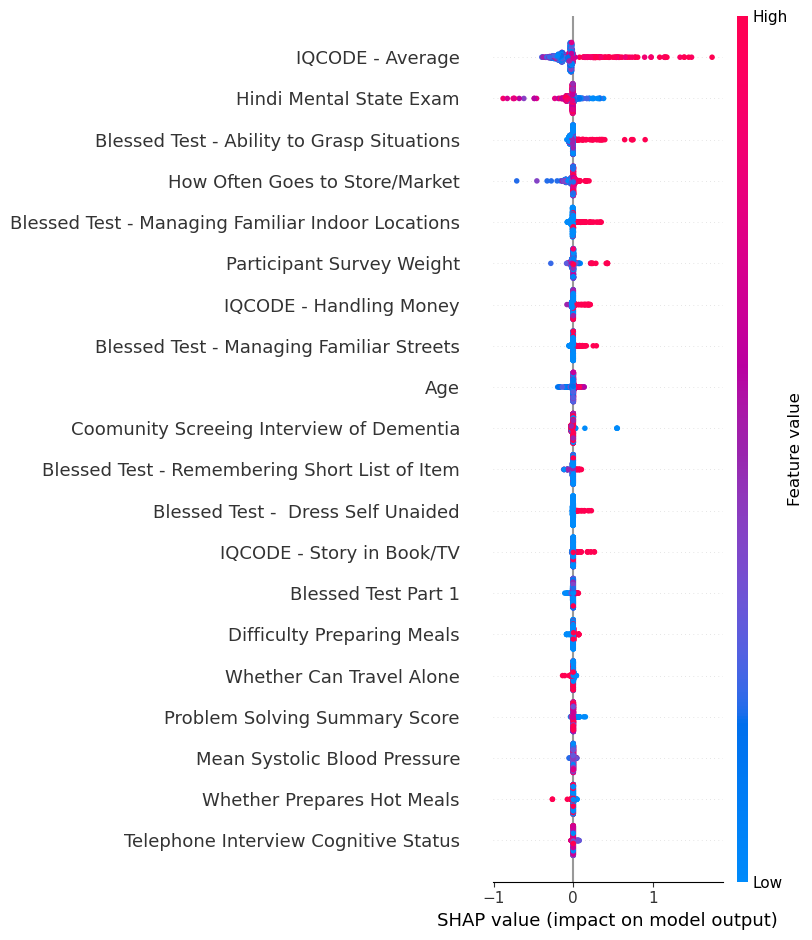


Threshold to Maximise Youden’s Index = 0.08

| Metric | Result |
| --- | --- |
| Accuracy | 0.96 |
| Sensitivity | 1.00 |
| Specificity | 0.80 |
| Area Under the Curve | 0.96 |
| Youden’s Index* | 0.80 |

* Defined as (Sensitivity + Specificity) - 1

Rater 4


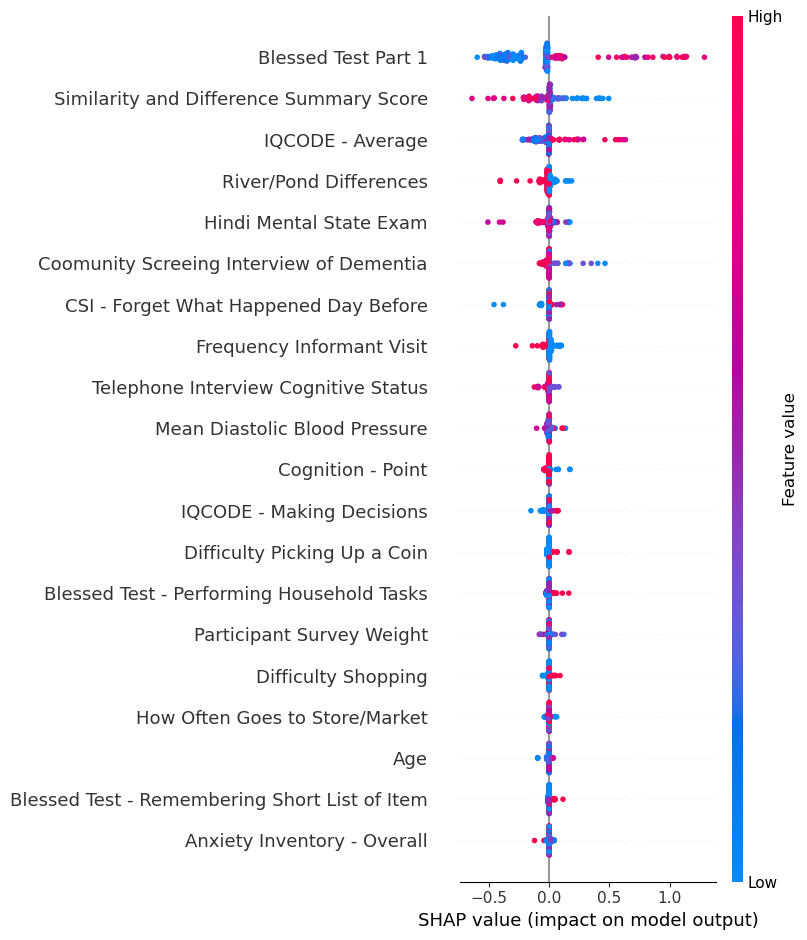


Threshold to Maximise Youden’s Index = 0.21

| Metric | Result |
| --- | --- |
| Accuracy | 0.92 |
| Sensitivity | 0.92 |
| Specificity | 0.92 |
| Area Under the Curve | 0.95 |
| Youden’s Index* | 0.84 |

* Defined as (Sensitivity + Specificity) - 1

Rater 5


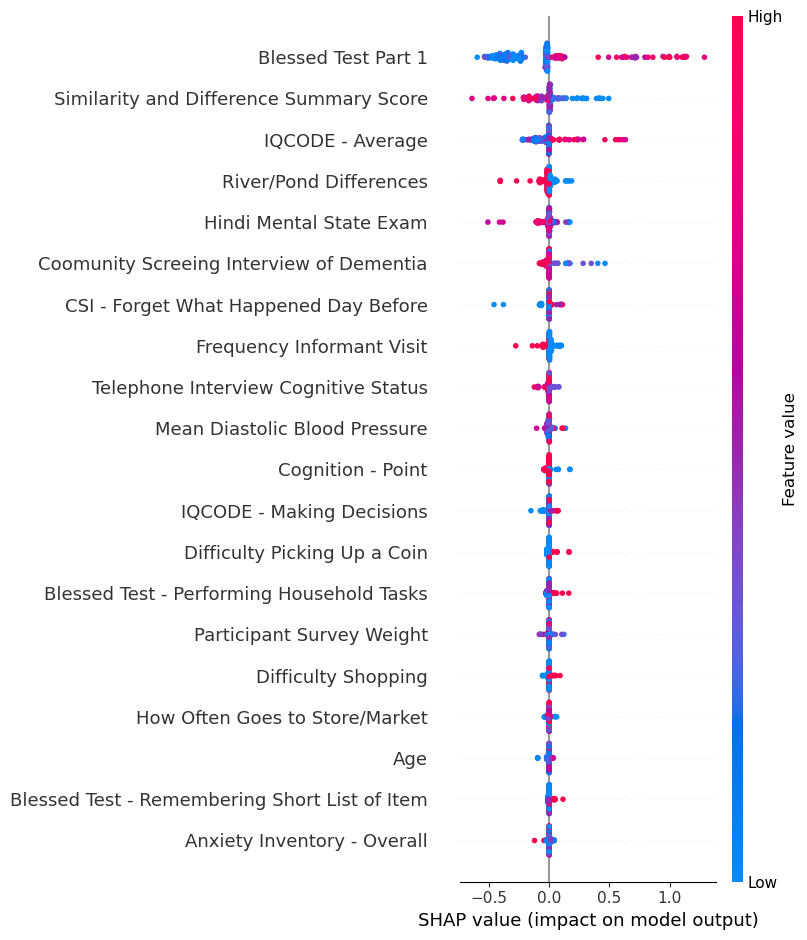


Threshold to Maximise Youden’s Index = 0.13

| Metric | Result |
| --- | --- |
| Accuracy | 0.91 |
| Sensitivity | 0.81 |
| Specificity | 0.92 |
| Area Under the Curve | 0.94 |
| Youden’s Index* | 0.73 |

* Defined as (Sensitivity + Specificity) - 1

Rater 6


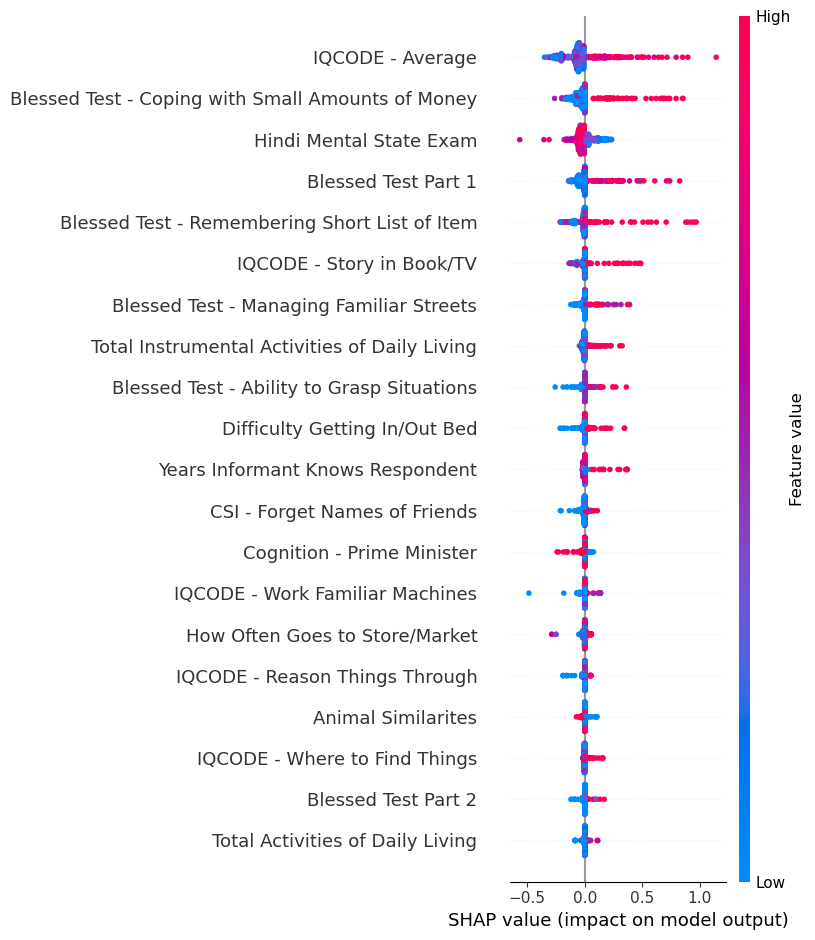


Threshold to Maximise Youden’s Index = 0.16

| Metric | Result |
| --- | --- |
| Accuracy | 0.93 |
| Sensitivity | 0.78 |
| Specificity | 0.94 |
| Area Under the Curve | 0.93 |
| Youden’s Index* | 0.72 |

* Defined as (Sensitivity + Specificity) - 1

Rater 7


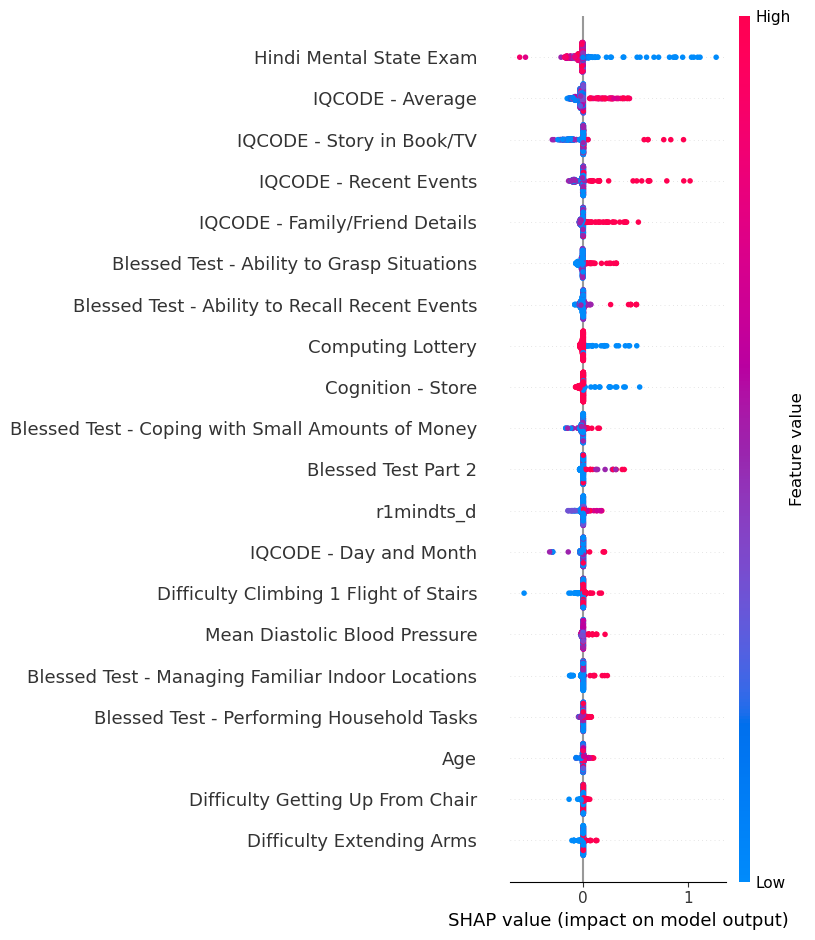


Threshold to Maximise Youden’s Index = 0.09

| Metric | Result |
| --- | --- |
| Accuracy | 0.91 |
| Sensitivity | 0.93 |
| Specificity | 0.91 |
| Area Under the Curve | 0.95 |
| Youden’s Index* | 0.84 |

* Defined as (Sensitivity + Specificity) - 1

Rater 8


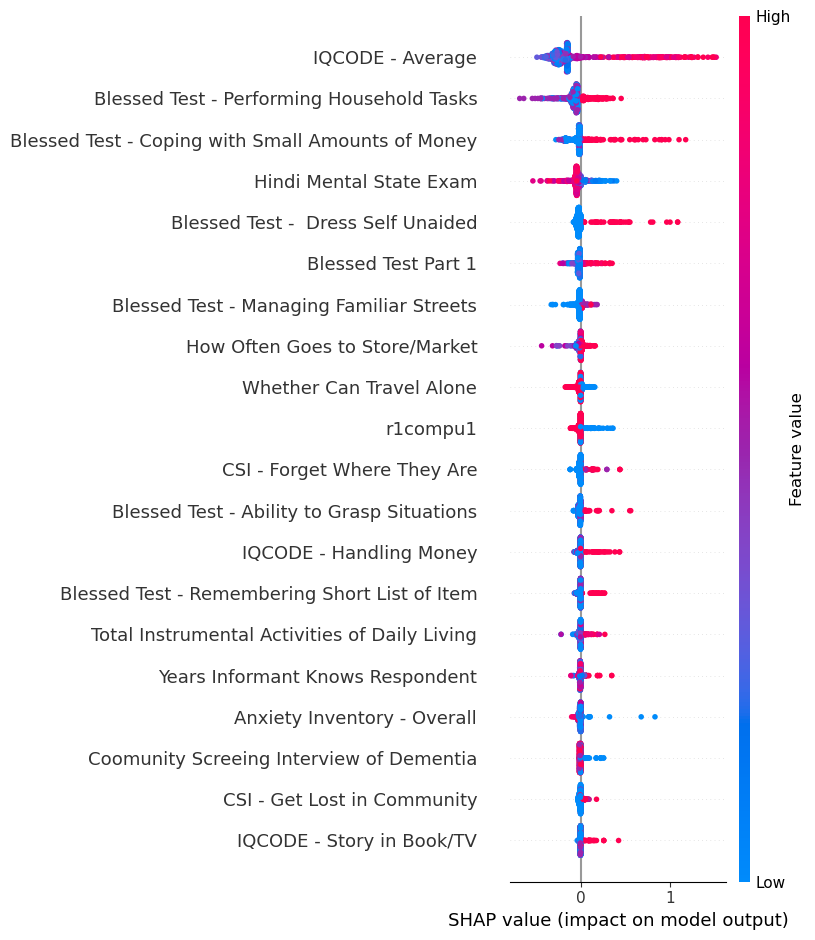


Threshold to Maximise Youden’s Index = 0.13

| Metric | Result |
| --- | --- |
| Accuracy | 0.89 |
| Sensitivity | 0.84 |
| Specificity | 0.89 |
| Area Under the Curve | 0.93 |
| Youden’s Index* | 0.73 |

* Defined as (Sensitivity + Specificity) - 1

Rater 9


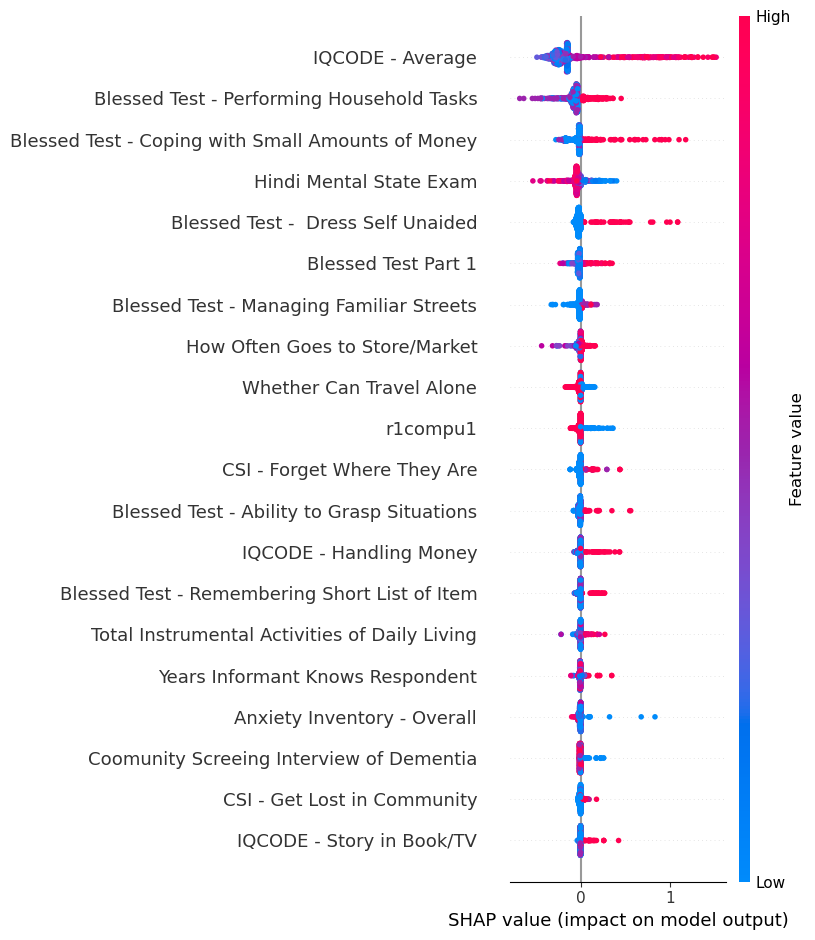


Threshold to Maximise Youden’s Index = 0.09

| Metric | Result |
| --- | --- |
| Accuracy | 0.91 |
| Sensitivity | 0.67 |
| Specificity | 0.92 |
| Area Under the Curve | 0.78 |
| Youden’s Index* | 0.59 |

* Defined as (Sensitivity + Specificity) - 1
