# Supplementary material for: Dementia ascertainment in India and development of nation‐specific cutoffs: A machine learning and diagnostic analysis
Source: Alzheimers Dement (Amst). 2025 Mar 28;17(1):e70049. doi: 10.1002/dad2.70049 (PMC11952995; doi:10.1002/dad2.70049)
Supplement: Supplementary file 5 — Supporting Information [file DAD2-17-e70049-s007.docx]

Supplemental File 4: Optimal cutoff (IQCODE 3.8; HMSE 25) results across various demographics using complete Hindi Mental State Examination scores. Each demographic group is listed first with table of metrics below

Overall:

| Metric | Result |
| --- | --- |
| Accuracy | 0.84 |
| Sensitivity | 0.90 (0.85 – 0.94) |
| Specificity | 0.84 (0.82 – 0.85) |
| Youden’s Index* | 0.74 (0.67 – 0.79) |

() contains 95% Confidence Interval; * Defined as (Sensitivity + Specificity) – 1

Female:

| Metric | Result |
| --- | --- |
| Accuracy | 0.81 |
| Sensitivity | 0.92 (0.85 – 0.97) |
| Specificity | 0.81 (0.78 – 0.83) |
| Youden’s Index* | 0.73 (0.69 – 0.77) |

() contains 95% Confidence Interval; * Defined as (Sensitivity + Specificity) – 1

Male:

| Metric | Result |
| --- | --- |
| Accuracy | 0.89 |
| Sensitivity | 0.88 (0.77 – 0.96) |
| Specificity | 0.89 (0.87 – 0.91) |
| Youden’s Index* | 0.77 (0.69 – 0.84) |

() contains 95% Confidence Interval; * Defined as (Sensitivity + Specificity) – 1

Low Education (< 6 years):

| Metric | Result |
| --- | --- |
| Accuracy | 0.80 |
| Sensitivity | 0.90 (0.86 – 0.96) |
| Specificity | 0.79 (0.76 – 0.81) |
| Youden’s Index* | 0.68 (0.66 – 0.72) |

() contains 95% Confidence Interval; * Defined as (Sensitivity + Specificity) – 1

High Education (>= 6 years):

| Metric | Result |
| --- | --- |
| Accuracy | 0.96 |
| Sensitivity | 0.90 (0.70 – 1.00) |
| Specificity | 0.96 (0.95 – 0.98) |
| Youden’s Index* | 0.87 (0.69 – 1.00) |

() contains 95% Confidence Interval; * Defined as (Sensitivity + Specificity) – 1

Illiterate:

| Metric | Result |
| --- | --- |
| Accuracy | 0.78 |
| Sensitivity | 0.90 (0.83 – 0.94) |
| Specificity | 0.77 (0.75 – 0.79) |
| Youden’s Index* | 0.67 (0.63 – 0.70) |

() contains 95% Confidence Interval; * Defined as (Sensitivity + Specificity) – 1

Literate:

| Metric | Result |
| --- | --- |
| Accuracy | 0.95 |
| Sensitivity | 0.94 (0.82 – 1.00) |
| Specificity | 0.95 (0.93 – 0.96) |
| Youden’s Index* | 0.89 (0.79 – 0.99) |

() contains 95% Confidence Interval; * Defined as (Sensitivity + Specificity) – 1

Rural:

| Metric | Result |
| --- | --- |
| Accuracy | 0.81 |
| Sensitivity | 0.89 (0.83 – 0.95) |
| Specificity | 0.81 (0.79 – 0.83) |
| Youden’s Index* | 0.70 (0.66 – 0.74) |

() contains 95% Confidence Interval; * Defined as (Sensitivity + Specificity) – 1

Urban:

| Metric | Result |
| --- | --- |
| Accuracy | 0.92 |
| Sensitivity | 0.93 (0.82 – 1.00) |
| Specificity | 0.92 (0.90 – 0.93) |
| Youden’s Index* | 0.85 (0.77 – 0.93) |

() contains 95% Confidence Interval; * Defined as (Sensitivity + Specificity) – 1
