# Supplementary material for: Dementia ascertainment in India and development of nation‐specific cutoffs: A machine learning and diagnostic analysis
Source: Alzheimers Dement (Amst). 2025 Mar 28;17(1):e70049. doi: 10.1002/dad2.70049 (PMC11952995; doi:10.1002/dad2.70049)
Supplement: Supplementary file 6 — Supporting Information [file DAD2-17-e70049-s006.docx]

Supplemental File 5: Figures show beeswarm plots depicting variable importance as measured by SHAP (Shapley Additive Explanations) values for participants subset by different demographic groups. For example, Rater 1 shows the variable importance for illiterate participants. Following the plot is a table of metrics showing the performance of the machine learning algorithm in this demographic group.

SHAP Value Beeswarm Plot for Illiterate Participants


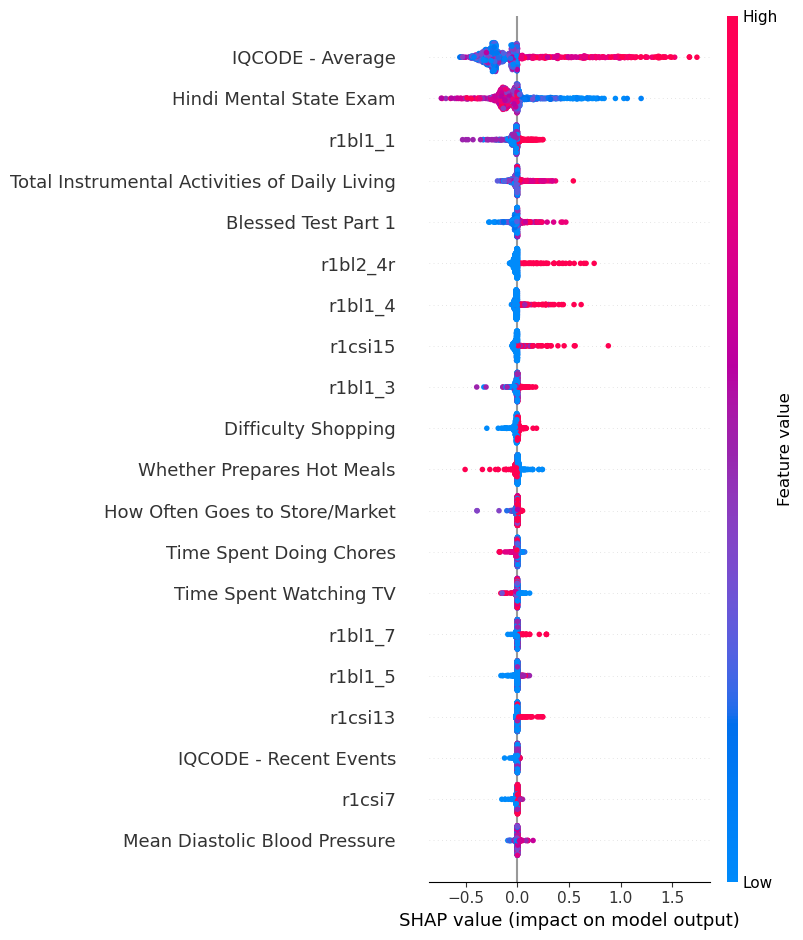


Threshold to Maximise Youden’s Index = 0.21

Performance metrics for machine learning algorithm for illiterate participants

| Metric | Result |
| --- | --- |
| Accuracy | 0.93 |
| Sensitivity | 0.82 |
| Specificity | 0.95 |
| Area Under the Curve | 0.91 |
| Youden’s Index* | 0.77 |

* Defined as (Sensitivity + Specificity) - 1

SHAP Value Beeswarm Plot for Participants in Rural Communities


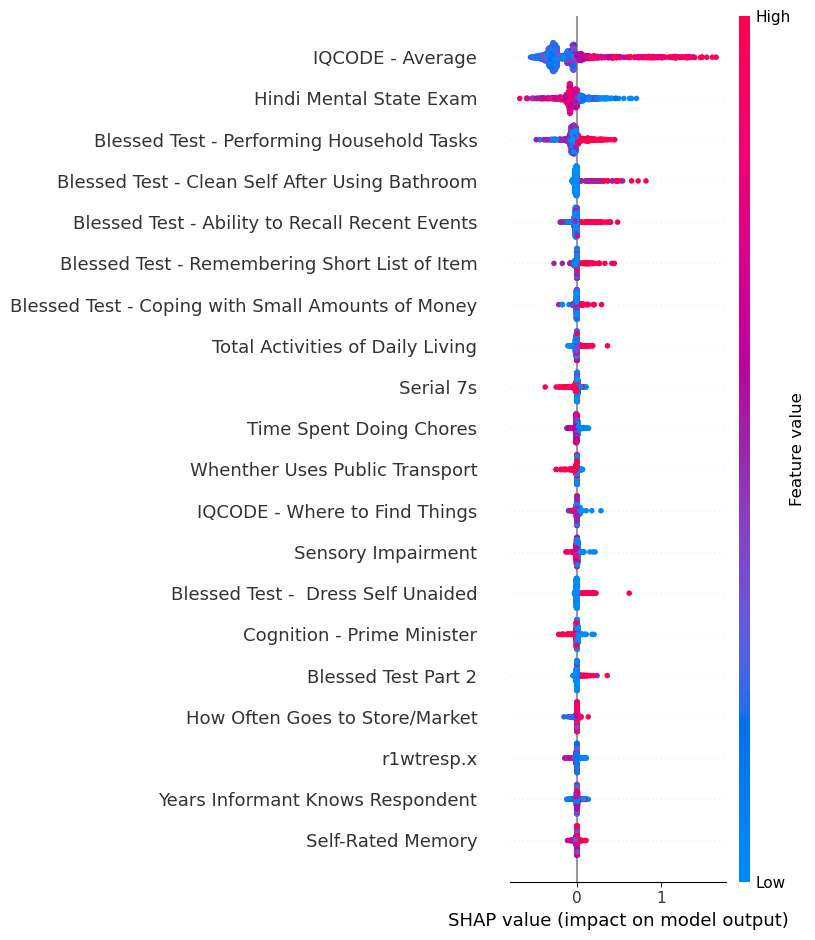


Threshold to Maximise Youden’s Index = 0.15

Performance metrics for machine learning algorithm for participants in rural communities

| Metric | Result |
| --- | --- |
| Accuracy | 0.92 |
| Sensitivity | 0.91 |
| Specificity | 0.92 |
| Area Under the Curve | 0.96 |
| Youden’s Index* | 0.83 |

* Defined as (Sensitivity + Specificity) - 1

SHAP Value Beeswarm Plot for Participants with Low Education (< 6 years)


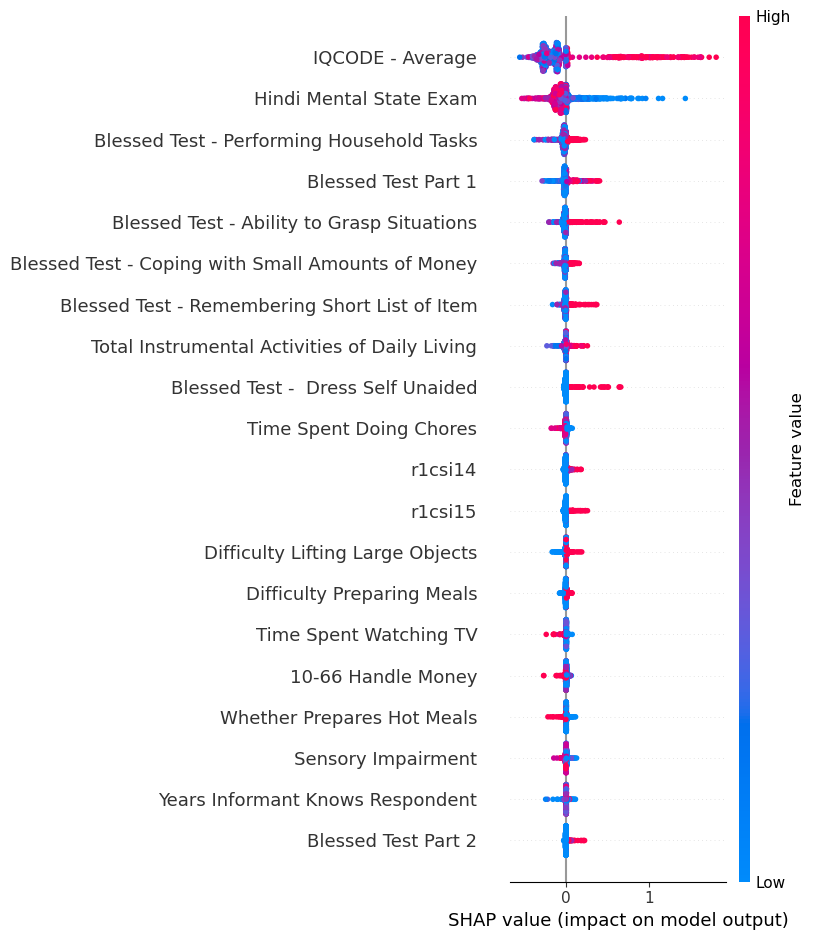


Threshold to Maximise Youden’s Index = 0.11

Performance metrics for machine learning algorithm for participants with low education (< 6 years)

| Metric | Result |
| --- | --- |
| Accuracy | 0.87 |
| Sensitivity | 0.94 |
| Specificity | 0.86 |
| Area Under the Curve | 0.95 |
| Youden’s Index* | 0.80 |

* Defined as (Sensitivity + Specificity) - 1
