# Supplementary material for: Dementia ascertainment in India and development of nation‐specific cutoffs: A machine learning and diagnostic analysis
Source: Alzheimers Dement (Amst). 2025 Mar 28;17(1):e70049. doi: 10.1002/dad2.70049 (PMC11952995; doi:10.1002/dad2.70049)
Supplement: Supplementary file 7 — Supporting Information [file DAD2-17-e70049-s005.docx]

Table of cut-off scores and metrics for dementia screening with IQCODE and HMSE on the LASI-DAD dataset

All participants had a complete HMSE

| Cut-off IQCODE |  | Cut-off HMSE | | | | | | | | | | |
| --- | --- | --- | --- | --- | --- | --- | --- | --- | --- | --- | --- | --- |
| IQCODE | Metric | hmse20 | hmse21 | hmse22 | hmse23 | hmse24 | hmse25 | hmse26 | hmse27 | hmse28 | hmse29 | hmse30 |
| IQCODE3 | Accuracy | 0.80 | 0.76 | 0.73 | 0.69 | 0.65 | 0.59 | 0.54 | 0.47 | 0.40 | 0.33 | 0.27 |
| IQCODE3 | Sensitivity | 0.80 | 0.83 | 0.87 | 0.89 | 0.92 | 0.94 | 0.96 | 0.98 | 0.98 | 0.99 | 0.99 |
| IQCODE3 | Specificity | 0.80 | 0.76 | 0.72 | 0.68 | 0.63 | 0.57 | 0.51 | 0.44 | 0.36 | 0.28 | 0.23 |
| IQCODE3 | AUC | 0.80 | 0.79 | 0.80 | 0.79 | 0.77 | 0.76 | 0.74 | 0.71 | 0.67 | 0.64 | 0.61 |
| IQCODE3 | Distance | 0.28 | 0.29 | 0.31 | 0.34 | 0.38 | 0.43 | 0.49 | 0.56 | 0.64 | 0.72 | 0.77 |
| IQCODE3 | Youdens_Index | 0.61 | 0.59 | 0.59 | 0.57 | 0.55 | 0.51 | 0.47 | 0.42 | 0.34 | 0.28 | 0.22 |
| IQCODE3.1 | Accuracy | 0.81 | 0.78 | 0.74 | 0.71 | 0.66 | 0.61 | 0.56 | 0.51 | 0.44 | 0.38 | 0.34 |
| IQCODE3.1 | Sensitivity | 0.80 | 0.83 | 0.87 | 0.89 | 0.92 | 0.94 | 0.96 | 0.98 | 0.98 | 0.99 | 0.99 |
| IQCODE3.1 | Specificity | 0.81 | 0.77 | 0.74 | 0.69 | 0.65 | 0.59 | 0.54 | 0.48 | 0.41 | 0.35 | 0.30 |
| IQCODE3.1 | AUC | 0.81 | 0.80 | 0.80 | 0.79 | 0.78 | 0.77 | 0.75 | 0.73 | 0.70 | 0.67 | 0.65 |
| IQCODE3.1 | Distance | 0.27 | 0.28 | 0.29 | 0.32 | 0.36 | 0.41 | 0.46 | 0.52 | 0.59 | 0.65 | 0.70 |
| IQCODE3.1 | Youdens_Index | 0.62 | 0.60 | 0.61 | 0.59 | 0.57 | 0.54 | 0.50 | 0.45 | 0.39 | 0.34 | 0.29 |
| IQCODE3.2 | Accuracy | 0.83 | 0.80 | 0.77 | 0.74 | 0.70 | 0.66 | 0.62 | 0.58 | 0.54 | 0.50 | 0.48 |
| IQCODE3.2 | Sensitivity | 0.80 | 0.82 | 0.86 | 0.89 | 0.91 | 0.93 | 0.95 | 0.97 | 0.98 | 0.98 | 0.98 |
| IQCODE3.2 | Specificity | 0.83 | 0.80 | 0.77 | 0.73 | 0.69 | 0.65 | 0.60 | 0.56 | 0.51 | 0.47 | 0.45 |
| IQCODE3.2 | AUC | 0.81 | 0.81 | 0.81 | 0.81 | 0.80 | 0.79 | 0.78 | 0.76 | 0.74 | 0.73 | 0.72 |
| IQCODE3.2 | Distance | 0.26 | 0.27 | 0.27 | 0.29 | 0.32 | 0.36 | 0.40 | 0.44 | 0.49 | 0.53 | 0.55 |
| IQCODE3.2 | Youdens_Index | 0.63 | 0.62 | 0.63 | 0.62 | 0.60 | 0.58 | 0.55 | 0.53 | 0.49 | 0.46 | 0.43 |
| IQCODE3.3 | Accuracy | 0.84 | 0.81 | 0.79 | 0.76 | 0.72 | 0.68 | 0.65 | 0.61 | 0.57 | 0.54 | 0.52 |
| IQCODE3.3 | Sensitivity | 0.79 | 0.81 | 0.85 | 0.88 | 0.90 | 0.93 | 0.94 | 0.96 | 0.97 | 0.97 | 0.97 |
| IQCODE3.3 | Specificity | 0.84 | 0.81 | 0.78 | 0.75 | 0.71 | 0.67 | 0.63 | 0.59 | 0.55 | 0.52 | 0.49 |
| IQCODE3.3 | AUC | 0.82 | 0.81 | 0.82 | 0.81 | 0.81 | 0.80 | 0.79 | 0.77 | 0.76 | 0.74 | 0.73 |
| IQCODE3.3 | Distance | 0.26 | 0.27 | 0.26 | 0.28 | 0.31 | 0.34 | 0.38 | 0.41 | 0.45 | 0.48 | 0.51 |
| IQCODE3.3 | Youdens_Index | 0.63 | 0.62 | 0.63 | 0.63 | 0.61 | 0.59 | 0.57 | 0.55 | 0.52 | 0.48 | 0.46 |
| IQCODE3.4 | Accuracy | 0.86 | 0.84 | 0.82 | 0.80 | 0.77 | 0.74 | 0.71 | 0.68 | 0.65 | 0.63 | 0.62 |
| IQCODE3.4 | Sensitivity | 0.78 | 0.80 | 0.85 | 0.87 | 0.89 | 0.92 | 0.93 | 0.95 | 0.96 | 0.96 | 0.96 |
| IQCODE3.4 | Specificity | 0.87 | 0.84 | 0.82 | 0.79 | 0.76 | 0.73 | 0.69 | 0.67 | 0.64 | 0.61 | 0.60 |
| IQCODE3.4 | AUC | 0.82 | 0.82 | 0.83 | 0.83 | 0.83 | 0.82 | 0.81 | 0.81 | 0.80 | 0.79 | 0.78 |
| IQCODE3.4 | Distance | 0.26 | 0.25 | 0.24 | 0.24 | 0.26 | 0.28 | 0.31 | 0.34 | 0.37 | 0.39 | 0.40 |
| IQCODE3.4 | Youdens_Index | 0.65 | 0.65 | 0.67 | 0.66 | 0.66 | 0.65 | 0.63 | 0.62 | 0.59 | 0.57 | 0.56 |
| IQCODE3.5 | Accuracy | 0.88 | 0.86 | 0.84 | 0.83 | 0.80 | 0.78 | 0.75 | 0.73 | 0.71 | 0.70 | 0.69 |
| IQCODE3.5 | Sensitivity | 0.78 | 0.80 | 0.85 | 0.87 | 0.89 | 0.92 | 0.93 | 0.94 | 0.95 | 0.95 | 0.95 |
| IQCODE3.5 | Specificity | 0.88 | 0.86 | 0.84 | 0.82 | 0.80 | 0.77 | 0.74 | 0.72 | 0.70 | 0.68 | 0.67 |
| IQCODE3.5 | AUC | 0.83 | 0.83 | 0.84 | 0.85 | 0.85 | 0.85 | 0.84 | 0.83 | 0.82 | 0.82 | 0.81 |
| IQCODE3.5 | Distance | 0.25 | 0.24 | 0.22 | 0.22 | 0.23 | 0.24 | 0.27 | 0.29 | 0.31 | 0.32 | 0.33 |
| IQCODE3.5 | Youdens_Index | 0.66 | 0.67 | 0.69 | 0.69 | 0.69 | 0.69 | 0.67 | 0.66 | 0.65 | 0.63 | 0.62 |
| IQCODE3.6 | Accuracy | 0.89 | 0.87 | 0.86 | 0.84 | 0.82 | 0.80 | 0.78 | 0.76 | 0.74 | 0.72 | 0.72 |
| IQCODE3.6 | Sensitivity | 0.77 | 0.80 | 0.84 | 0.86 | 0.89 | 0.91 | 0.92 | 0.93 | 0.94 | 0.94 | 0.94 |
| IQCODE3.6 | Specificity | 0.90 | 0.88 | 0.86 | 0.84 | 0.82 | 0.79 | 0.77 | 0.75 | 0.72 | 0.71 | 0.70 |
| IQCODE3.6 | AUC | 0.83 | 0.84 | 0.85 | 0.85 | 0.85 | 0.85 | 0.84 | 0.84 | 0.83 | 0.83 | 0.82 |
| IQCODE3.6 | Distance | 0.25 | 0.24 | 0.22 | 0.21 | 0.22 | 0.23 | 0.25 | 0.26 | 0.28 | 0.29 | 0.30 |
| IQCODE3.6 | Youdens_Index | 0.67 | 0.67 | 0.70 | 0.70 | 0.70 | 0.70 | 0.69 | 0.68 | 0.67 | 0.65 | 0.65 |
| IQCODE3.7 | Accuracy | 0.91 | 0.89 | 0.88 | 0.87 | 0.85 | 0.84 | 0.81 | 0.80 | 0.78 | 0.77 | 0.76 |
| IQCODE3.7 | Sensitivity | 0.76 | 0.79 | 0.83 | 0.85 | 0.88 | 0.90 | 0.91 | 0.92 | 0.93 | 0.93 | 0.93 |
| IQCODE3.7 | Specificity | 0.92 | 0.90 | 0.89 | 0.87 | 0.85 | 0.83 | 0.81 | 0.79 | 0.77 | 0.76 | 0.75 |
| IQCODE3.7 | AUC | 0.84 | 0.84 | 0.86 | 0.86 | 0.86 | 0.87 | 0.86 | 0.85 | 0.85 | 0.84 | 0.84 |
| IQCODE3.7 | Distance | 0.25 | 0.23 | 0.21 | 0.20 | 0.19 | 0.20 | 0.21 | 0.22 | 0.24 | 0.25 | 0.26 |
| IQCODE3.7 | Youdens_Index | 0.68 | 0.69 | 0.71 | 0.72 | 0.73 | 0.73 | 0.72 | 0.71 | 0.70 | 0.69 | 0.68 |
| IQCODE3.8 | Accuracy | 0.92 | 0.90 | 0.90 | 0.88 | 0.87 | 0.85 | 0.83 | 0.82 | 0.80 | 0.79 | 0.79 |
| IQCODE3.8 | Sensitivity | 0.76 | 0.79 | 0.83 | 0.85 | 0.88 | 0.90 | 0.91 | 0.92 | 0.93 | 0.93 | 0.93 |
| IQCODE3.8 | Specificity | 0.93 | 0.91 | 0.90 | 0.89 | 0.87 | 0.85 | 0.83 | 0.81 | 0.80 | 0.79 | 0.78 |
| IQCODE3.8 | AUC | 0.85 | 0.85 | 0.86 | 0.87 | 0.87 | 0.88 | 0.87 | 0.87 | 0.86 | 0.86 | 0.85 |
| IQCODE3.8 | Distance | 0.25 | 0.23 | 0.20 | 0.19 | 0.18 | 0.18 | 0.19 | 0.20 | 0.22 | 0.23 | 0.23 |
| IQCODE3.8 | Youdens_Index | 0.69 | 0.70 | 0.73 | 0.74 | 0.74 | 0.75 | 0.74 | 0.73 | 0.72 | 0.71 | 0.71 |
| IQCODE3.9 | Accuracy | 0.92 | 0.91 | 0.90 | 0.89 | 0.88 | 0.87 | 0.85 | 0.85 | 0.83 | 0.83 | 0.82 |
| IQCODE3.9 | Sensitivity | 0.71 | 0.73 | 0.76 | 0.79 | 0.81 | 0.84 | 0.85 | 0.85 | 0.86 | 0.86 | 0.86 |
| IQCODE3.9 | Specificity | 0.94 | 0.92 | 0.91 | 0.90 | 0.88 | 0.87 | 0.85 | 0.85 | 0.83 | 0.82 | 0.82 |
| IQCODE3.9 | AUC | 0.82 | 0.83 | 0.84 | 0.84 | 0.85 | 0.85 | 0.85 | 0.85 | 0.85 | 0.84 | 0.84 |
| IQCODE3.9 | Distance | 0.30 | 0.28 | 0.25 | 0.23 | 0.22 | 0.21 | 0.21 | 0.21 | 0.22 | 0.22 | 0.23 |
| IQCODE3.9 | Youdens_Index | 0.64 | 0.65 | 0.68 | 0.69 | 0.70 | 0.71 | 0.70 | 0.70 | 0.69 | 0.68 | 0.68 |
| IQCODE4 | Accuracy | 0.94 | 0.93 | 0.93 | 0.92 | 0.91 | 0.90 | 0.89 | 0.89 | 0.88 | 0.87 | 0.87 |
| IQCODE4 | Sensitivity | 0.66 | 0.67 | 0.71 | 0.72 | 0.74 | 0.76 | 0.77 | 0.78 | 0.79 | 0.79 | 0.79 |
| IQCODE4 | Specificity | 0.96 | 0.95 | 0.94 | 0.93 | 0.92 | 0.91 | 0.90 | 0.89 | 0.88 | 0.88 | 0.88 |
| IQCODE4 | AUC | 0.81 | 0.81 | 0.82 | 0.83 | 0.83 | 0.84 | 0.84 | 0.84 | 0.84 | 0.83 | 0.83 |
| IQCODE4 | Distance | 0.34 | 0.33 | 0.30 | 0.28 | 0.27 | 0.25 | 0.25 | 0.24 | 0.24 | 0.24 | 0.24 |
| IQCODE4 | Youdens_Index | 0.61 | 0.62 | 0.65 | 0.66 | 0.66 | 0.68 | 0.67 | 0.67 | 0.67 | 0.67 | 0.67 |
| IQCODE4.1 | Accuracy | 0.94 | 0.94 | 0.94 | 0.93 | 0.92 | 0.92 | 0.91 | 0.90 | 0.89 | 0.89 | 0.89 |
| IQCODE4.1 | Sensitivity | 0.64 | 0.66 | 0.69 | 0.71 | 0.72 | 0.75 | 0.76 | 0.76 | 0.77 | 0.77 | 0.77 |
| IQCODE4.1 | Specificity | 0.96 | 0.96 | 0.95 | 0.94 | 0.93 | 0.93 | 0.92 | 0.91 | 0.90 | 0.90 | 0.90 |
| IQCODE4.1 | AUC | 0.80 | 0.81 | 0.82 | 0.82 | 0.83 | 0.84 | 0.84 | 0.84 | 0.84 | 0.84 | 0.83 |
| IQCODE4.1 | Distance | 0.36 | 0.34 | 0.31 | 0.30 | 0.28 | 0.26 | 0.26 | 0.25 | 0.25 | 0.25 | 0.25 |
| IQCODE4.1 | Youdens_Index | 0.60 | 0.61 | 0.64 | 0.65 | 0.66 | 0.67 | 0.67 | 0.67 | 0.67 | 0.67 | 0.67 |
| IQCODE4.2 | Accuracy | 0.95 | 0.94 | 0.94 | 0.94 | 0.93 | 0.93 | 0.92 | 0.92 | 0.91 | 0.91 | 0.91 |
| IQCODE4.2 | Sensitivity | 0.57 | 0.59 | 0.61 | 0.63 | 0.64 | 0.67 | 0.67 | 0.67 | 0.68 | 0.68 | 0.68 |
| IQCODE4.2 | Specificity | 0.97 | 0.97 | 0.96 | 0.96 | 0.95 | 0.95 | 0.94 | 0.93 | 0.93 | 0.93 | 0.92 |
| IQCODE4.2 | AUC | 0.77 | 0.78 | 0.79 | 0.79 | 0.80 | 0.81 | 0.81 | 0.80 | 0.81 | 0.80 | 0.80 |
| IQCODE4.2 | Distance | 0.43 | 0.42 | 0.39 | 0.38 | 0.36 | 0.34 | 0.33 | 0.33 | 0.33 | 0.33 | 0.33 |
| IQCODE4.2 | Youdens_Index | 0.54 | 0.55 | 0.57 | 0.58 | 0.59 | 0.61 | 0.61 | 0.61 | 0.61 | 0.61 | 0.61 |
| IQCODE4.3 | Accuracy | 0.95 | 0.95 | 0.94 | 0.94 | 0.94 | 0.93 | 0.93 | 0.92 | 0.92 | 0.92 | 0.92 |
| IQCODE4.3 | Sensitivity | 0.54 | 0.56 | 0.59 | 0.60 | 0.62 | 0.64 | 0.65 | 0.65 | 0.66 | 0.66 | 0.66 |
| IQCODE4.3 | Specificity | 0.97 | 0.97 | 0.97 | 0.96 | 0.96 | 0.95 | 0.95 | 0.94 | 0.94 | 0.93 | 0.93 |
| IQCODE4.3 | AUC | 0.76 | 0.77 | 0.78 | 0.78 | 0.79 | 0.80 | 0.80 | 0.80 | 0.80 | 0.80 | 0.80 |
| IQCODE4.3 | Distance | 0.46 | 0.44 | 0.42 | 0.40 | 0.38 | 0.36 | 0.35 | 0.35 | 0.35 | 0.35 | 0.35 |
| IQCODE4.3 | Youdens_Index | 0.52 | 0.53 | 0.55 | 0.56 | 0.57 | 0.59 | 0.60 | 0.59 | 0.59 | 0.59 | 0.59 |
| IQCODE4.4 | Accuracy | 0.95 | 0.95 | 0.95 | 0.94 | 0.94 | 0.94 | 0.94 | 0.93 | 0.93 | 0.93 | 0.93 |
| IQCODE4.4 | Sensitivity | 0.51 | 0.53 | 0.54 | 0.56 | 0.58 | 0.60 | 0.61 | 0.61 | 0.62 | 0.62 | 0.62 |
| IQCODE4.4 | Specificity | 0.98 | 0.98 | 0.97 | 0.97 | 0.96 | 0.96 | 0.96 | 0.96 | 0.95 | 0.95 | 0.95 |
| IQCODE4.4 | AUC | 0.75 | 0.75 | 0.76 | 0.76 | 0.77 | 0.78 | 0.78 | 0.78 | 0.79 | 0.78 | 0.78 |
| IQCODE4.4 | Distance | 0.49 | 0.47 | 0.46 | 0.44 | 0.42 | 0.40 | 0.39 | 0.39 | 0.39 | 0.39 | 0.39 |
| IQCODE4.4 | Youdens_Index | 0.49 | 0.51 | 0.52 | 0.53 | 0.54 | 0.56 | 0.57 | 0.56 | 0.57 | 0.57 | 0.57 |
| IQCODE4.5 | Accuracy | 0.95 | 0.95 | 0.95 | 0.95 | 0.94 | 0.94 | 0.94 | 0.94 | 0.94 | 0.94 | 0.94 |
| IQCODE4.5 | Sensitivity | 0.41 | 0.41 | 0.43 | 0.44 | 0.46 | 0.48 | 0.49 | 0.49 | 0.50 | 0.50 | 0.50 |
| IQCODE4.5 | Specificity | 0.99 | 0.98 | 0.98 | 0.98 | 0.97 | 0.97 | 0.97 | 0.97 | 0.97 | 0.96 | 0.96 |
| IQCODE4.5 | AUC | 0.70 | 0.70 | 0.71 | 0.71 | 0.71 | 0.73 | 0.73 | 0.73 | 0.73 | 0.73 | 0.73 |
| IQCODE4.5 | Distance | 0.59 | 0.59 | 0.57 | 0.56 | 0.55 | 0.52 | 0.51 | 0.51 | 0.51 | 0.51 | 0.51 |
| IQCODE4.5 | Youdens_Index | 0.39 | 0.40 | 0.41 | 0.42 | 0.43 | 0.45 | 0.46 | 0.46 | 0.46 | 0.46 | 0.46 |
| IQCODE4.6 | Accuracy | 0.95 | 0.95 | 0.95 | 0.95 | 0.95 | 0.95 | 0.94 | 0.94 | 0.94 | 0.94 | 0.94 |
| IQCODE4.6 | Sensitivity | 0.37 | 0.38 | 0.40 | 0.41 | 0.42 | 0.43 | 0.44 | 0.44 | 0.45 | 0.45 | 0.45 |
| IQCODE4.6 | Specificity | 0.99 | 0.99 | 0.98 | 0.98 | 0.98 | 0.98 | 0.98 | 0.97 | 0.97 | 0.97 | 0.97 |
| IQCODE4.6 | AUC | 0.68 | 0.68 | 0.69 | 0.69 | 0.70 | 0.70 | 0.71 | 0.71 | 0.71 | 0.71 | 0.71 |
| IQCODE4.6 | Distance | 0.63 | 0.62 | 0.60 | 0.59 | 0.58 | 0.57 | 0.56 | 0.56 | 0.55 | 0.55 | 0.55 |
| IQCODE4.6 | Youdens_Index | 0.36 | 0.37 | 0.38 | 0.39 | 0.40 | 0.41 | 0.41 | 0.41 | 0.42 | 0.42 | 0.42 |
| IQCODE4.7 | Accuracy | 0.95 | 0.95 | 0.95 | 0.95 | 0.95 | 0.95 | 0.95 | 0.95 | 0.95 | 0.95 | 0.95 |
| IQCODE4.7 | Sensitivity | 0.32 | 0.33 | 0.33 | 0.34 | 0.36 | 0.37 | 0.37 | 0.37 | 0.38 | 0.38 | 0.38 |
| IQCODE4.7 | Specificity | 0.99 | 0.99 | 0.99 | 0.99 | 0.99 | 0.98 | 0.98 | 0.98 | 0.98 | 0.98 | 0.98 |
| IQCODE4.7 | AUC | 0.65 | 0.66 | 0.66 | 0.66 | 0.67 | 0.68 | 0.68 | 0.68 | 0.68 | 0.68 | 0.68 |
| IQCODE4.7 | Distance | 0.68 | 0.67 | 0.67 | 0.66 | 0.64 | 0.63 | 0.63 | 0.63 | 0.62 | 0.62 | 0.62 |
| IQCODE4.7 | Youdens_Index | 0.31 | 0.32 | 0.32 | 0.33 | 0.34 | 0.35 | 0.36 | 0.36 | 0.36 | 0.36 | 0.36 |
| IQCODE4.8 | Accuracy | 0.95 | 0.95 | 0.95 | 0.95 | 0.95 | 0.95 | 0.95 | 0.95 | 0.95 | 0.95 | 0.95 |
| IQCODE4.8 | Sensitivity | 0.28 | 0.28 | 0.28 | 0.29 | 0.30 | 0.30 | 0.30 | 0.30 | 0.31 | 0.31 | 0.31 |
| IQCODE4.8 | Specificity | 0.99 | 0.99 | 0.99 | 0.99 | 0.99 | 0.99 | 0.99 | 0.99 | 0.99 | 0.99 | 0.99 |
| IQCODE4.8 | AUC | 0.63 | 0.63 | 0.64 | 0.64 | 0.65 | 0.64 | 0.64 | 0.64 | 0.65 | 0.65 | 0.65 |
| IQCODE4.8 | Distance | 0.72 | 0.72 | 0.72 | 0.71 | 0.70 | 0.70 | 0.70 | 0.70 | 0.69 | 0.69 | 0.69 |
| IQCODE4.8 | Youdens_Index | 0.27 | 0.27 | 0.28 | 0.28 | 0.29 | 0.29 | 0.29 | 0.29 | 0.30 | 0.30 | 0.30 |
| IQCODE4.9 | Accuracy | 0.95 | 0.95 | 0.95 | 0.95 | 0.95 | 0.95 | 0.95 | 0.95 | 0.95 | 0.95 | 0.95 |
| IQCODE4.9 | Sensitivity | 0.15 | 0.15 | 0.15 | 0.16 | 0.16 | 0.16 | 0.16 | 0.16 | 0.16 | 0.16 | 0.16 |
| IQCODE4.9 | Specificity | 1.00 | 1.00 | 1.00 | 1.00 | 1.00 | 0.99 | 0.99 | 0.99 | 0.99 | 0.99 | 0.99 |
| IQCODE4.9 | AUC | 0.58 | 0.58 | 0.58 | 0.58 | 0.58 | 0.58 | 0.58 | 0.58 | 0.58 | 0.58 | 0.58 |
| IQCODE4.9 | Distance | 0.85 | 0.85 | 0.85 | 0.84 | 0.84 | 0.84 | 0.84 | 0.84 | 0.84 | 0.84 | 0.84 |
| IQCODE4.9 | Youdens_Index | 0.15 | 0.15 | 0.15 | 0.16 | 0.16 | 0.16 | 0.16 | 0.16 | 0.16 | 0.16 | 0.16 |
| IQCODE5 | Accuracy | 0.94 | 0.94 | 0.94 | 0.94 | 0.94 | 0.94 | 0.94 | 0.94 | 0.94 | 0.94 | 0.94 |
| IQCODE5 | Sensitivity | 0.00 | 0.00 | 0.00 | 0.00 | 0.00 | 0.00 | 0.00 | 0.00 | 0.00 | 0.00 | 0.00 |
| IQCODE5 | Specificity | 1.00 | 1.00 | 1.00 | 1.00 | 1.00 | 1.00 | 1.00 | 1.00 | 1.00 | 1.00 | 1.00 |
| IQCODE5 | AUC | 0.50 | 0.50 | 0.50 | 0.50 | 0.50 | 0.50 | 0.50 | 0.50 | 0.50 | 0.50 | 0.50 |
| IQCODE5 | Distance | 1.00 | 1.00 | 1.00 | 1.00 | 1.00 | 1.00 | 1.00 | 1.00 | 1.00 | 1.00 | 1.00 |
| IQCODE5 | Youdens_Index | 0.00 | 0.00 | 0.00 | 0.00 | 0.00 | 0.00 | 0.00 | 0.00 | 0.00 | 0.00 | 0.00 |
| Comparison Outcome is Clinical Dementia Rating where > 0.5 signifies dementia | | | | | | | | | | | | |
| Youden's Index = Sensitivity + Specificity - 1. Higher is better | | | | | | | | | | | | |
| Distance = distance from upper left corner or perfect discrimination. Lower is better | | | | | | | | | | | | |
